# Creating local estimates from a population health survey: practical application of small area estimation methods

**DOI:** 10.3934/publichealth.2020034

**Published:** 2020-06-22

**Authors:** Diane Hindmarsh, David Steel

**Affiliations:** 1 Bureau of Health Information, Level 2, 1 Reserve Road St Leonards, NSW, Australia; 2 National Institute for Applied Statistics Research Australia, University of Wollongong, Wollongong, NSW, Australia

**Keywords:** SAE, small area estimation, survey, health, risk factors, population health, smoking rates, risk alcohol drinking

## Abstract

Regular health surveys can produce reliable estimates at higher geographic levels but not for small areas. Alternatives are to aggregate data over several years or use model-based methods. We created and evaluated model-based estimates for four health-related outcomes by gender, for 153 Local Government Areas using data from the New South Wales Population Health Survey. The evaluation examined evidence on bias and determined the covariates available and appropriate for each outcome variable. The evaluation considered the likely precision of the resulting estimates. The bias and precision of results for single years (2006–2008) for each outcome variable using six covariate specifications were compared with direct survey estimates based on a single year's data and those obtained by aggregating over seven years. A practical issue is how to choose covariates to include in the models as the best covariate specification varies between outcome variables. Model-based results had median root mean squared errors between 3.3% and 5.5% (max 5.2% and 11.3% respectively) and median relative root mean squared errors between 6.8% and 24.5% (max 11.7% and 41.5% respectively). The model-based estimates were unbiased compared with direct estimates based on one or seven years of data and when aggregated to a point where direct estimates were reliable. The bias and reliability assessment process provides a way for policymakers to have confidence in model-based estimates.

## 1. Introduction

Information for each local area is important for policy development and evaluation for the local area. The problem is that often there is a lack of up-to-date information available at the local area, and the information that is available is updated infrequently. The reason for this dearth of information is that it is very expensive to have sufficient sample for each local area. So, policy makers usually have to rely on data collated over many years in order to get sufficient sample size [1], use estimates based on larger regional areas where surveys can provide estimates with reasonable standard errors [2], or only present results for local areas with sufficient sample size, suppressing the remainder [3],[4].

An alternative is to use model-based small area estimation (SAE) methods [5], but these can be seen as too complex to easily integrate into current data processes. That is, they are unable to be operationalised in routine analysis of a large repeated population health survey to produce timely estimates the way that direct estimates can be. For methods to be accepted and adopted as a routine approach they must be able to be understood and applied by analysts or epidemiologists working in the population health survey program.

This paper describes a practical example of the development and evaluation of robust and readily applied SAE methods to data from a state-based population health survey to provide local area estimates using the empirical best predictor (EBP) associated with a logistic mixed model. We consider the key decisions and evaluations that have to make along the way, such as whether the random effect in the EBP model is needed, and how to determine the covariates in the model. In addition, we assess the reliability of the resulting estimates and their bias compared with the unbiased but unreliable direct estimates. This paper aims to make SAE methods accessible so that they can be applied more regularly in routine analysis of large-scale population health surveys to produce current and timely estimates for local areas.

We demonstrate the methods using data from the NSW Population Health Survey (PHS), a dual frame computer assisted telephone interview (CATI) survey run by the Ministry of Health NSW. We focus on four key dichotomous outcome variables that differ in prevalence and in the level of between-area variance: “Current Smoking (SMK)”, “Risk Alcohol Consumption (ALC)”, “Overweight or obese (BMI)” and “Have difficulties getting health care when needed (HDIFF)”. At the time of this study, when estimates at the local area level were requested, direct estimates based on seven years of data were supplied, with suppression of results for local areas where there were fewer than 300 respondents in that 7-year period. Therefore, we concentrate on comparing model-based estimates with direct estimates based on seven years of data, as well as comparing aggregated model-based estimates with direct estimates based on one year of data at a level where direct estimates are regularly published.

## 2. Materials and method

### 2.1. NSW population health survey

The study used unit-record data from the NSW PHS. This survey is designed to produce annual estimates for the health administrative areas. The number of administrative areas has changed over time; between 2005 and 2010 there were eight such areas [6]. During this same period there were 153 local government areas (LGAs), and the aim was to create estimates at the LGA level.

Although data were available for the period 2002 to 2008 (inclusive), the modelling was undertaken on the data from 2006 to 2008. The full seven years of data were used as comparators to the model-based results, as this was the usual method of providing LGA-level results at the time of this study. Detailed information regarding sample selection, weighting, stratification and other aspects of the survey are provided elsewhere [7]–[10].

During the telephone-based interview, respondents are asked to name their LGA, postcode and/or the suburb in which they live. If LGA is not provided, then it is assigned using postcode or suburb data. In order to align with the 2006 census data, the 2006 LGA boundary definitions were used for this study [11]. The unincorporated area in far west NSW was included with the 152 LGAs so that full coverage of NSW was obtained, resulting in 153 areas. Over the study period 2800 (3.6%) of respondents could not be assigned to LGAs. These were excluded from the study, leaving 75,175 observations, with between 7,783 and 12,736 in individual years. Not all questions were asked in 2007, which reduces the number of respondents for some questions.

#### 2.1.1. Direct estimates and estimates of variance

Direct LGA-level estimates were obtained for each of the four outcome variables, by sex, for each of the three most recent years of data available (2006–2008). Direct estimates based on small sample sizes are unreliable, however, they are asymptotically unbiased. Direct estimates were also obtained for each individual LGA by aggregating all seven years of survey data—the method used to provide LGA-level estimates at the time. The SAS SURVEYMEAN procedure was used to calculate the direct estimates and associated estimated standard errors accounting for the sample design. LGA was included as a domain. Health area, sex and age group were included as strata variables and the post-stratification weight was included. Estimates were adjusted so that the population-weighted average agreed with the published estimate for that year for each outcome variable, by sex.

This paper makes the assumption that estimates of variability based on direct estimates are unbiased and therefore we use the term standard error (SE) and relative standard error (RSE) when assessing variability. Model-based estimates may be biased, and therefore, to indicate that the estimate of variability includes both variance and bias terms, we refer to estimates of variability as mean square errors (MSEs), estimated root mean square errors (RMSEs) and relative root mean square errors (RRMSEs).

#### 2.1.2. Explanatory variables available from the NSW population health survey

Model-based SAE methods exploit the relationship between the outcome variables and covariate information estimated from the survey, in combination with population level covariate information from a population census or other sources. The following variables were available from the survey data for possible inclusion as explanatory variables in the models to create LGA level estimates:

- Variables derived from the sampling process: health area, number of people in the household;
- Demographic variables collected in the survey: age group, sex, highest level of education, country of birth, language usually spoken at home, marital status, employment status, pension status (for over 65-year olds), income in broad bands, private health cover status;
- Contextual variables for the LGA derived from variables collected in the survey: quintile of relative socioeconomic disadvantage (IRSD) and remoteness index (ARIA score). These were based on ABS data for these indices, with data provided by NSW Health.

#### 2.1.3. Source of LGA-level covariate data

While the coefficients of model parameters are estimated solely using survey data, model-based estimates require population proportions for each covariate for each small area. Gender-specific LGA population proportions were sourced from the Basic Community Profile of the 2006 Census of Population and Housing [12] for the majority of covariates. Person-level information on pensions was obtained by combining data from the national regional profile [13] and the Social Health Atlas [14]. Person-level estimates of private health insurance cover by LGA were obtained from the Social Health Atlas [14]. See [15] for more information.

### 2.2. Small area estimator—the empirical best predictor

Although other options were tested in early work [15], we concentrate on the most promising model: a unit (person) level generalised linear mixed model with a logit link (1), see [5].

$$logit\left(y_{ig}\right)=\;x_{ig}^{\prime }\beta +\upsilon_{g}$$ (1)

where *υ_g_* are independent *N*(0, *σ*_2_), *i* = 1…*n_g_*, *g* = 1…*G*

In model (1), *y_ig_* is the response of the *i^th^* person in the *g^th^* LGA and *n_g_* is the sample size in the *g^th^* LGA, *x_ig_* is the vector of covariate values from the survey, *β* is the vector of regression coefficients and *υ_g_* is a random effect reflecting area level effect.

When the sampling fraction is small the estimator of the mean for area *g*, ${\tilde{\bar{\theta }}}_{g}$ is,

$${\tilde{\bar{\theta }}}_{g}=\frac{exp\left({{\bar{X}}^{\prime }}_{g}\hat{\beta }+{\hat{\upsilon }}_{g}\right)}{1+exp\left({{\bar{X}}^{\prime }}_{g}\hat{\beta }+{\hat{\upsilon }}_{g}\right)}$$ (2)

where *X_g_* is the vector of population proportions of the covariates, $\hat{\upsilon }$ is the estimated random effect for the *g^th^* LGA and $\hat{\beta }$ is the vector of estimated regression coefficients [15].

The estimator in equation (2) is known as the Empirical Best Predictor (EBP). The synthetic estimator is obtained by omitting the $\hat{\upsilon }$. Inclusion of the random effect term, when important, should improve the estimates and the validity of the estimated RMSEs. The estimate of the regression coefficient $\hat{\beta }$ and the random effect $\hat{\upsilon }$ can be obtained using the unit level survey data with area indicators using any statistical software that can fit model (1). This study used the GLIMMIX procedure in SAS because routine survey analysis is undertaken in SAS by NSW Ministry of Health.

To create small area estimates the EBP, the population means of the covariates, *X_g_* need to be available for each area. When covariates can be displayed as population proportions, the small area estimates are easily obtained using (2). The unit level model is fit with each covariate as a set of indicator variables (0 if not applicable, 1 if applicable). The small area estimate is obtained using (2), replacing the indicator variables with the appropriate area-level proportion. Determining which covariate variables are available and deciding which ones to use in the model is a key practical step in developing and applying model-based SAE methods and is in section 2.2.4.

Synthetic estimates and EBPs were obtained for each of the four outcome variables, by sex, using six covariate specifications. As is typical in survey analysis, the variables included in the survey weighting were included as covariates in the model-building process, where statistically significant, therefore the survey weights were not included in the analyses [17]. The synthetic estimator was obtained in order to assess their bias (see section 2.3), and to examine estimated RMSEs when the random effect is zero, in which case the EBP becomes the synthetic estimator.

#### 2.2.1. Estimated MSE of the EBP

Although it is relatively easy to create a small area estimate, the estimation of the MSE of the EBP estimator is complicated, usually requiring Monte Carlo methods or iterative steps using Maximum Penalised Quasi-Likelihood (MPQL) together with REML [5]. However, the variance created by the GLIMMIX procedure includes the most important components of the MSE. In addition, preliminary work showed that the estimated MSEs from the SAS GLIMMIX procedure were similar to those obtained using a parametric bootstrap procedure [15]. Therefore, we used the variance from SAS GLIMMIX to obtain estimated RMSEs and associated RRMSEs. Out-of-sample areas, where *n_g_* = 0, were allocated the maximum RMSE for in-sample areas, based on preliminary work that showed that the only time when the SAS-based RMSE was under-estimated was when the sample size was zero for an area [15],[18].

#### 2.2.2. Estimation of intraclass correlation coefficient

The intraclass correlation (ICC) for the logistic model was estimated using the latent variable formulation [19],

$${\hat{\rho }}_{\left(logit\right)}=\frac{{\hat{\sigma }}_{\upsilon }^{2}}{{\hat{\sigma }}_{\upsilon }^{2}+\frac{\pi^{2}}{3}}$$ (3)

where ${\hat{\sigma }}_{\upsilon }^{2}$ is the estimated variance of the LGA random effects, and *π* = 22/7.

#### 2.2.3. Estimating model fit at unit- and small area level

The level of variability explained by the models at the individual level is measured using the adjusted *R*^2^ [20]. The more relevant aspect of fit when estimating at the small area level is the variability explained at the area level. However, unlike when undertaking simulation studies, with real data there is no ‘truth’. Therefore, we devised an area-level pseudo-*R*^2^ (4) as a way of measuring area-level fit. It aims to capture the reduction in unexplained variation using the mean of the model-based predictions for each sampled individual in an area, when compared with a null model.

$$R_{LGA}^{2}=1-\frac{\sum_{g}n_{g}^{\text{*}}\;{\left({\bar{y}}_{g}-n_{g}^{-1}\sum_{i\in g}{\hat{p}}_{ig}\right)}^{2}}{\sum_{g}n_{g}^{\text{*}}\;{\left({\bar{y}}_{g}-n_{g}^{-1}\sum_{i\in g}{\hat{p}}_{ig}{}^{o}\right)}^{2}}$$ (4)

where ${\hat{p}}_{ig}$ is the model-based estimate for the *i^th^* person from the *g^th^* area, ${\hat{p}}_{ig}{}^{o}$ is the corresponding null model estimate, which was defined as the state mean for the particular year and sex, calculated without weighting, *y_g_* was defined as the sample mean for the *g^th^* area (without weights) and $n_{g}^{\text{*}}$ is the actual sample size at the LGA level used in the calculation of the numerator. The sample size was included in the calculation of pseudo-*R*^2^ to allow for the fact that models could have been based on different sample sizes. Gelman et al. [21] have also derived a pseudo-*R*^2^, however they do so to avoid an issue where the *R*^2^ in the Bayesian paradigm may be greater than unity. In the current case it is used to estimate the fit at the level of reporting, rather than at the unit record level.

#### 2.2.4. Model development

We considered six covariate specifications (see Table 1). Models were estimated separately for male and females using data for each year from 2006 to 2008.

**Table 1.** Covariate specifications studied in this analysis.

Abbreviation	Covariates included	Comments
Covariates consistent between outcome variables		
Null		Intercept only model
Age	10-year age group only	
ONS	10-year age group, AHS and quintile of IRSD	Global model that includes contextual effects. Based on model used for small area estimates by the Office of National Statistics (ONS) in the UK [22]
Global	All covariates included in any of the outcome-specific Comm models (see below for Comm model)	Consistent Global model includes unit-level and contextual effects (quintile of IRSD and AHS)
Covariates in model differ between outcome variables		
Specific (Spec)	Outcome and model-specific. Covariates that are significant following stepwise regression for the specific sex/ outcome/ year	Theoretically this is the best possible model, but it is inconsistent over time, so not particularly useful when developing methods for routine reporting. Model selection was undertaken without inclusion of the random effect
Common (Comm)	Covariates that are significant in the majority of years for that sex/outcome	Outcome-specific model; i.e. same within each outcome by sex and year

Four of the models have the same covariate specification for all outcomes while the other two were specific to the outcome and sex. While outcome-specific models may be the best by construction, they lead to different sets of covariates being used in different years for the same outcome variable, so the models with consistent variables were included to see if a more parsimonious model could be used.

The variables in the outcome-specific (Spec) models were based on results of a backward selection process using the LOGISTIC procedure in SAS. This was undertaken separately for each outcome variable-sex-year combination. The statistical significance level used to keep variables in the model set at 0.05. Whilst the LOGISTIC procedure does not include a random effect term, it was considered suitable for the purposes of covariate selection.

The covariates included in the common model were the covariates that occurred in at least half of the Specific models for that outcome-sex grouping when the results for the 7 years were considered, including logistic and linear models. Results for linear models are not included in this paper (see [15]).

The models were restricted to main effects because of the challenge of accessing population data at the LGA level of the quality required to include interaction terms. Cross-tabulated data, by LGA and sex will include small cell sizes and the ABS modifies small numbers in such cells to assure confidentiality [14],[22].

As mentioned in section 2.2., LGA level estimates are obtained by substituting the proportions for the indicator variables in the EBP (2). This is easily achieved in SAS and other software by appending LGA-level population proportions to the dataset. As these records do not have observed values for the outcome variables, they are not included in the model creation. Despite this, because they are in the dataset, predicted values and associated measures of variability will be created. In SAS both synthetic and EBP estimates and their associated measures of variability can be obtained from a single run of the procedure. See Supplementary files for more information.

LGA-level estimates of health risk factors presented in the social health atlas [14] are based on a synthetic estimator. In order to determine whether the inclusion of the random effect is necessary, we include an analysis in section 3.3.3. that considers the impact of the random effect on the RMSEs. This may be considered unnecessary by those with strong familiarity with SAE methods, however it is worthwhile addressing this issue rather than making an assumption that the random effects are necessary.

All estimates were adjusted so that the weighted average of the 153 LGA-based estimates agreed with the published NSW rates for the appropriate year.

### 2.3. Evaluation of bias and precision

Evaluation of the methods considers whether the resultant estimates are reliable enough to be useful to inform policy and assist in developing healthier communities in NSW.

If the model is correctly specified, the resulting estimates will be unbiased, but in practice the model may be inadequate in some way and the resulting estimates may be biased, albeit with lower variability compared with direct estimates.

For measures of variability, we concentrated on the median and maximum RMSEs at LGA level, comparing them comparable statistics based on direct estimates, by sex and outcome. We compared the model-based estimates in three ways as follows.

#### 2.3.1. Comparison 1: model-based estimates v.s. direct estimates based on one year of data

Most direct estimates based on a single year of data are unreliable and unfit for reporting due to small sample size and therefore high SEs. Despite this, direct estimates are asymptotically unbiased [23], so they can still be used in assessing evidence of bias.

The first part of the comparison is to ensure that the difference between direct and modelled estimates decreased as sample size increased.

We used the method of Brown et al. [23] to assess bias. This method assesses whether the relationship between the synthetic estimates (as the independent variable) and the unbiased direct estimates (as the dependent variable) is compatible with the line of identity. According to [23], it is appropriate to use the synthetic estimator for this part of the evaluation because the expected value of the random effect is zero. We assessed this relationship on the original scale, weighted by sample size and when using the square root transformation for both sets of estimates. Another alternative is to assess correlation [25], however assessing conformity to the line of identity is a stronger test than testing that the two to have statistically significant correlation.

#### 2.3.2. Comparison 2: Comparison of aggregated model-based estimates v.s. direct estimates

We created a weighted mean of the model-based estimates, weighted by population size, at both the health area level and by quintile of IRSD. These are the geographic levels at which at which direct estimates are publicly reported.

Results for each of the four outcome variables were collated by gender and year and assessed as a single group for each outcome as otherwise the comparison had insufficient power. These comparisons include between 30 and 48 observations. The relevant comparisons were as follows:

- The proportion of aggregated model-based estimates falling within the 95% confidence interval around the direct estimate calculated at health area or by quintile of IRSD, and
- Whether the relationship between aggregated model-based estimates and direct estimates was consistent with the line of identity.

Both sets of estimates were transformed to the square root in order to take into account potential heteroscedasticity in estimated proportions.

#### 2.3.3. Comparison 3: Model-based estimates v.s. LGA-level direct estimates 7 years of data

This comparison used the most appropriate covariate specification given the responses to the previous two comparisons. It compares the model-based estimates and associated measures of variability against what was usually provided when LGA-level estimates were requested. We were particularly interested in the range in estimates, RMSE and RRMSE of model-based estimates compared with the direct estimates and associated SE and RSE. Both sets of estimates were adjusted so that the weighted average agreed to the reported state average for that year.

## 3. Results and discussion

In this section, we provide information on the data source and direct estimates for one outcome variable to explain why direct estimates are not appropriate for all LGAs, even when based on data aggregated over seven years. We consider development of model-based estimates, including consideration of how model complexity affects the differences in estimated RMSE between the EBP and synthetic estimators. We then consider how the model-based estimates compare against direct estimates using the three comparison types mentioned in section 2.3.3. Finally, we consider whether model-based estimates and associated RMSEs and RRMSEs achieve a desired level for use in practical settings.

### 3.1. NSW population health survey: sample sizes

The number of responses aggregated over the seven years ranged from 16 to 777 for males and 16 to 1178 for females (Table 2). It is not unusual for more females than males to respond to the NSW population health surveys [11].

The number of responses to individual questions was affected by a decision made in 2007 to reduce the burden on respondents [7]. Table 2 presents the sample size able to be used to obtain direct estimates for the BMI outcome variable. It shows that the impact was a reduction of about 10% compared with the total number of respondents. It reduces the number of LGAs with more than 300 responses from 38 to 31 for males and from 53 to 45 for females. The maximum sample sizes for SMK were slightly higher than for BMI, with 709 for males and 1080 for females (Table 3).

**Table 2.** Descriptive statistics for response numbers at LGA level, by sex. Overall numbers of responses and sample size applicable for BMI outcome variable, 2002–2008 combined.

	Gender	Min	Q1	Median	Q3	Max	Number of LGAs with	
							GT 150 responses	GT 300 responses
Number of respondents to surveys	Female	16	91	201	392	1178	88	53
	Male	16	69	126	283	777	70	38
Sample size for BMI question	Female	14	79	170	334	1047	81	45
	Male	13	62	112	253	694	66	31

Although larger sample sizes are available if results are reported at the person level, policy development is strengthened when based on gender-based results [24]. While person-level results cannot be split by gender unless they are provided in the first place, gender-based results can be combined to create person-level results.

#### 3.1.1. NSW population health survey: direct estimates and estimates of variance

Summary statistics for LGA-level direct estimates (DEs) of current smoking (SMK) rates, by gender, based on data aggregated data from 2002–2008 are presented in Table 3. This provides an example of the direct estimates for this aggregated period. The median standard error is less than 5%, with maxima between 13% and 14% for males and females respectively. The median relative standard errors (RSEs) are 23.1% and 20.8% for males and females respectively, but the maximum RSEs are over 85% for both males and females. Direct estimates from eight LGAs would have had estimated RSEs of greater than 50% for males; another 58 LGAs have estimated RSEs of more than 25%. For females, 7 LGAs have estimated RSEs of greater than 50% and 47 LGAs have RSEs of more than 25%.

**Table 3.** Summary statistics for direct estimates, SMK, by sex, 2002–2008 combined.

Gender	Type	Min	Median	Mean	Max
Male	Sample size	15	113	182.4	709
	DE	6.4%	19.2%	20.1%	38.4%
	SE	1.5%	4.0%	4.9%	13.7%
	RSE	7.9%	23.1%	24.3%	96.4%
Female	Sample size	15	181	263.5	1080
	DE	3.3%	17.0%	17.0%	49.6%
	SE	1.3%	3.3%	3.8%	13.2%
	RSE	7.4%	20.8%	23.7%	86.6%

The Australian Bureau of Statistics suppresses estimates with RSEs greater than 50% and urges caution when using estimates with RSE more than 25% [12]. Based on the results in Table 3, the direct estimates for a considerable number of LGAs would be suppressed or presented but flagged as being of insufficient quality using these criteria even when results are based on 7 years of survey data.

### 3.2. Model-based small area estimates

We now turn to model-based methods. We provide information on the covariates that were included in the more complex model specifications before looking at the effect of model complexity and the effect of the inclusion of the random effect on the estimated RMSE.

#### 3.2.1. Model development: determining covariates in the specific models

Whilst the null, age and ONS models use the same covariates for all outcome variables (Table 1), the Specific and Common models relied on the results of the backward selection process. Information on covariates included in specific models is provided in Supplementary Table A. Table 4 shows the terms that were included in the Common models. The Global specification also used the same covariates for all outcome variables: it included all covariates presented in Table 4.

**Table 4.** Variables that were included in the common model, by outcome and sex.

Covariate	Categories	Reference Category	SMK		ALC		HDIFF		BMI	
			M	F	M	F	M	F	M	F
10yr age grp	7	75+	Y	Y	Y	Y	Y	Y	Y	Y
Health Area	8	Greater Western AHS			Y	Y	Y	Y	Y	Y
Marital status	4	Single	Y	Y		Y		Y	Y	Y
Education	4	Higher education	Y	Y		Y	Y	Y		Y
Country of birth	2	Australian-born		Y	Y	Y			Y	Y
Quintile of IRSD	5	Most disadvantaged		Y			Y	Y	Y	Y
Private health	2	No private health cover	Y	Y			Y	Y		Y
Employment status	2	No job			Y	Y	Y			Y
Language spoken at home	2	English			Y	Y				
Remoteness	3	Outer regional and remote					Y	Y		
Pension	2	No pension				Y				Y
Household size	5	Single person household				Y				

Model selection for the Specific model did not include the random effect term. Indeed, when the random effect is included, the statistically significant terms in the model can change. The inclusion of the random effect term is justified on the basis that under the model the effect is expected to be present and is needed to reflect local effects not captured by the covariates available.

It is useful to consider what variables ended up in common models (Table 4). The only covariate that is included in all of these models is age group. Health area is included in the models for all but SMK, whilst remoteness is only included for difficulties getting health care when needed, for which remoteness would be expected to be important. The models for females tended to have more terms than males. For instance, there is an effect of marital status in females for ALC, but not for males; pension and household size are only included in the Common models for females.

#### 3.2.2. Effect of the random effect term on estimated RMSE: EBP v.s. Synthetic estimators

Table 5 shows the median and maximum estimated RMSEs for EBP and synthetic estimates to highlight the fact that the random effect term increases the estimated RMSE due to inclusion of the area-level variance. Effectively the random effect term protects against over-confidence in the model.

The estimated RMSE for more complex synthetic models approaches that of the EBP but is still lower than the estimated RMSE of the EBP. It is well documented that the random effect term acts as a proxy for unknown between area variability, so it is of no surprise that the estimated RMSE of the EBP is higher than for the synthetic estimator, which assumes no area effects. The impact of ignoring the area random effects reduces when a more complex model is fitted.

**Table 5.** Maximum and Median estimated RMSE of EBP and logistic synthetic estimates, by sex, covariate specification group and outcome variable.

Sex	Outcome variable	Covariate specification group*	Median		Maximum	
			EBP	Synth	EBP	Synth
Male	BMI	null, age	3.8%	1.0%	4.1%	1.2%
		Other	3.7%	2.9%	4.5%	3.8%
	ALC	null, age	5.5%	1.1%	6.2%	1.3%
		Other	4.0%	3.1%	4.6%	3.7%
	HDIFF	null, age	6.1%	0.9%	11.5%	1.0%
		Other	5.5%	3.1%	9.1%	4.5%
	SMK	null, age	4.4%	1.0%	5.4%	1.1%
		Other	3.2%	2.2%	4.7%	3.2%
Female	BMI	null, age	5.2%	1.0%	5.9%	1.1%
		Other	3.6%	2.7%	4.2%	3.3%
	ALC	null, age	5.2%	0.9%	6.4%	1.1%
		Other	3.9%	2.6%	5.0%	3.5%
	HDIFF	null, age	6.8%	1.1%	14.6%	1.1%
		Other	6.7%	3.4%	10.9%	4.4%
	SMK	null, age	3.9%	0.7%	5.3%	0.9%
		Other	3.5%	1.9%	5.4%	2.8%

Note: * Null, age—averaged over the null and age-only models; Other—averaged over ONC, Specific, Global and common models.

#### 3.2.3. Estimated ICC of the logistic mixed model

Table 6 shows the estimated approximate ICC for the logistic mixed models for each of the outcome variables for 2008. A similar trend was observed in other years and are included in Supplementary Table B. ICC is lower for the more complex models, but even small levels of ICC have reasonable influence on the small area estimates, especially at small sample sizes [15]. Of the four outcome variables, HDIFF always had larger ICC values, suggesting that there is more between-area variability in this indicator than the other three that were studied.

**Table 6.** Estimated Approximate Intraclass correlation (ICC) of logistic mixed models, by covariate specification, outcome variable and sex, 2008.

Outcome variable	Sex	null	age	glob	ONS	comm	spec
BMI	Male	1.3%	1.2%	0.6%	1.2%	0.4%	0.1%
	Female	2.1%	2.5%	0.6%	0.9%	0.6%	0.8%
ALC	Male	1.6%	1.9%	0.0%	0.8%	0.1%	0.1%
	Female	2.0%	2.5%	0.2%	1.0%	0.3%	0.3%
HDIFF	Male	10.9%	11.1%	2.2%	3.3%	1.9%	1.9%
	Female	10.9%	12.3%	4.5%	5.3%	4.5%	4.5%
SMK	Male	1.5%	1.2%	0.4%	0.4%	0	0
	Female	3.9%	4.0%	2.1%	2.7%	2.3%	2.3%

Note: 0 indicates that the random error term was zero for these models; See [15] for similar results for 2006 and 2007.

HDIFF was also the only outcome variable for which the area level effect remains in the model all the time. For other outcomes, the area-level random effect term dropped out in approximately 10% of the models across the three years used in modelling. When the random effect term drops out the model-based estimates simply revert to the synthetic form. As Table 5 shows, the estimated RMSE of the synthetic estimator is still lower, but much closer to the RMSE of the EBP for the more complex models.

The EBP model does not always converge. The non-convergence occurred in 2007 on three occasions, once for each outcome variable except HDIFF (see Supplementary Table B). In all cases the other three complex models converged without any issues. In addition, at times the random effect may be zero, as mentioned earlier. Both of these indicate the importance of checking output and scrutinising analysis logs. We noted earlier that not all questions were asked in 2007, which may have caused the lack of convergence.

#### 3.2.4. Estimating the model fit of the EBP estimator

Small area estimation relies on exploiting strong relationships between the outcome and covariates in order to reduce the level of unexplained variability [5]. The unit level adjusted R-square values in Table 7 show that the models fitted to the unit level data explain a small amount of variability in the outcome variables, but this is measuring model fit at the individual level not the more relevant area level. At the area level there was approximately a 50% reduction in unexplained variation for HDIFF compared with the null model. The reduction in unexplained variation for the other outcomes was about 25%.

These pseudo-*R*^2^ values suggest that there is still a large amount of unexplained area level variability. It is interesting to assess model-based estimators against some form of standard, but analysis involving real rather that simulated data there is no gold standard. This is why the next section assesses bias, by analysing the difference between an asymptotically unbiased estimator and the expected value of the EBP.

**Table 7.** Unit level adjusted *R*^2^ and area-level pseudo-*R*^2^ values, averaged over years, by sex and outcome variable, specific (Spec) model.

	Male		Female	
	Unit level R^2^ (adj)	Area-level Pseudo R^2^	Unit level R^2^ (adj)	Area-level Pseudo R^2^
SMK	17%	25%	16%	24%
ALC	6%	22%	12%	31%
HDIFF	11%	43%	12%	51%
BMI	9%	22%	7%	40%

### 3.3. Assessment of bias

As mentioned in section 2.3., the assessment of bias included three specific comparisons: against direct estimates based on the same year of data; model-based estimates aggregated to higher levels of geography against direct estimates at the same level of geography; comparison against direct estimates based on seven years of data.

#### 3.3.1. Comparison of model-based results against direct estimates based on the same year of data

The Brown et al. method [23] showed that most covariate specifications could be considered consistent with the line of identity (or unbiased) against the theoretically unbiased direct estimates based on a single year of data (see Supplementary Table C for further information). The Age-only model was least consistent with the line of identity, although it was one of only two models showing consistency with the line of identity for SMK in females in 2006; the other was the common model. Agreement with the line of identity for BMI in males, 2008 required both direct and EBP estimates to be weighted. They were also unbiased for Age only and Specific covariate specifications when transformed to the square root scale.

**Figure 1.**
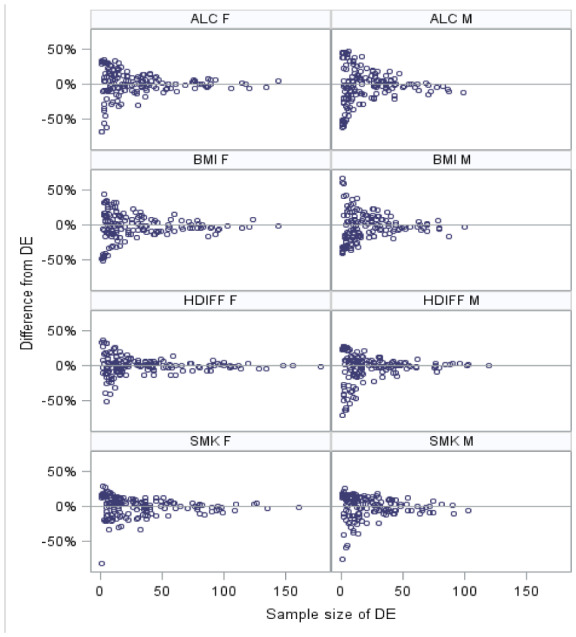
Difference between EBP and 1-year DE at LGA level, by outcome and gender, 2008.

While the direct estimates based on a single year of data are highly variable, they are unbiased and asymptotically approach the true value. Therefore, examining whether the comparison is consistent with the line of identity can provide one indication of bias. This comparison is the first of three that, together, help in deciding the appropriate model.

All model-based estimates approached the direct estimate as sample size increases. Figure 1 presents the result for the four outcome variables, by sex for 2008. The 2006 and 2007 results showed similar pattern.

#### 3.3.2. Comparison of aggregated EBP v.s. direct estimates

Table 8 shows the proportion of times the model-based EBP estimates aggregated to the health area level were located within the 95% confidence interval around the direct estimate. The null and age-only covariate specifications have poor coverage, with coverage less than 85% for ALC and BMI. Estimates for all the outcome variables using any of the four more complex models have coverage of 94% or more, with the exception of BMI with the global model.

This method of assessment is conservative as it ignores the variance associated with the model-based estimates. Adding margins of error to the differences between the EBP and aggregated estimates is complex. Even so, this simple test can build confidence in the model-based estimates for analysts who are wary of model-based methods.

When aggregated model-based results for the more complex covariate specifications are compared with direct estimates at the level of quintile of IRSD, at least 90% of aggregated estimates for ALC and BMI were located within the 95% confidence interval (Table 8); in comparison between 70% and 80% of aggregated model-based estimates for SMK and HDIFF were located within these 95% confidence limits. Although these are not as high as for the health area comparison. Data issues may have contributed to this result [15].

**Table 8.** Proportion of aggregated model-based estimates that lie within the 95% confidence interval around the direct estimate at health area level and by quintile of IRSD, by outcome and covariate specification.

	Null	Age	Global	ONS	Common	Specific	Average^
Health Area: approx * 48 observations per comparison							
ALC	81%	79%	95%	100%	96%	96%	97%
BMI	77%	77%	55%	96%	94%	96%	95%
HDIFF	87%	90%	100%	100%	100%	100%	100%
SMK	92%	90%	100%	100%	98%	98%	99%
Quintile of the index of socioeconomic disadvantage (IRSD): approx* 30 observations per comparison							
ALC	93%	93%	84%	97%	100%	100%	95%
BMI	67%	70%	88%	93%	90%	93%	91%
HDIFF	77%	73%	63%	80%	72%	70%	71%
SMK	73%	70%	83%	87%	80%	77%	82%

Note: * Sample sizes differ due to non-convergence for three sex-year models; ˆAverage is the average of Global, ONS, Common and Specific models.

The relatively low level of coverage of the 95% confidence intervals for the null and age-only models make them inappropriate as alternatives to the direct estimates. They are therefore not included in subsequent results.

When the aggregated model-based estimates were plotted against the direct estimates at these higher levels of geography, almost all were consistent with the line of identity. The only models that produced estimates were not consistent with the line of identity when assessed using the quintile of IRSD were ONS and Common for SMK, and Global for ALC. When aggregated to health area, Global and Specific models were not consistent with the line of identity for HDIFF and ONS model was not consistent for ALC.

#### 3.3.3. Determination of most appropriate covariate specification

The results to this point indicate that models need more than age and a random effect term. For determining which covariate specification is most appropriate, it appears to differ between outcome variable. However, each outcome variable was able to be associated with at least two covariate specifications that provided unbiased estimates that are consistent with direct estimates at the health area level. Of these, one was more appropriate than the other(s). For instance, for HDIFF the two unbiased models at the AHS level are ONS and Common. The Common model did not converge for females in 2007, so the preference is the ONS model. For ALC and SMK the choice is between a model specification that may change over time (Specific) and a more general model (the common model for ALC and Global model for SMK). In both cases it is suggested the more general model is the most useful option as it means the model does not change over time, which is an important practical consideration for routine production of SAEs.

Based on these findings, the covariates included in the final models for the four outcome variables are shown in Table 9. ALC was the only outcome where the model differed between males and females.

**Table 9.** Information on final models for the four outcome variables.

Outcome	Type of model	Variables included
ALC	Common	F: Age group, AHS, born in Australia, employment status, language spoken at home, aged pension status, household size, marital status, education level
		M: Age group, AHS, born in Australia, employment status, language spoken at home
BMI	ONS	Age group, AHS and quintile of IRSD
HDIFF	ONS	Age group, AHS and quintile of IRSD
SMK	Global	Age group, AHS, born in Australia, employment status, language spoken at home, aged pension status, household size, marital status, education level, IRSD, private health status, ARIA

#### 3.3.4. Comparison of final EBP estimates against direct estimates based on aggregated data

Various statistics are presented in Table 10 to compare the model-based estimates for 2008 using the final models given in Table 9 against the direct estimates based on the seven years of data.

**Table 10.** Various statistics of LGA-level direct estimates based on aggregated data and model-based estimates for 2008, by outcome and sex.

Outcome variable	Sex	Type of Estimate	Min	Q1	Median	Q3	Max
ALC	Male	DE0208	10.7%	37.6%	44.5%	49.4%	77.7%
		Model-based	22.7%	38.2%	43.8%	45.7%	49.2%
	Female	DE0208	8.5%	25.8%	30.3%	35.3%	57.5%
		Model-based	13.5%	29.3%	31.4%	32.8%	39.6%
BMI	Male	DE0208	36.4%	57.7%	63.4%	71.7%	94.0%
		Model-based	49.7%	59.2%	62.1%	65.9%	71.0%
	Female	DE0208	19.6%	44.4%	50.6%	55.7%	72.1%
		Model-based	34.4%	45.0%	50.7%	53.7%	60.4%
HDIFF	Male	DE0208	0.0%	11.9%	19.7%	28.1%	81.7%
		Model-based	5.9%	11.9%	22.6%	26.2%	36.6%
	Female	DE0208	4.3%	17.5%	30.0%	40.5%	79.5%
		Model-based	12.2%	19.1%	29.3%	37.5%	61.5%
SMK	Male	DE0208	6.5%	16.2%	19.7%	24.8%	39.4%
		Model-based	10.6%	15.1%	17.8%	20.9%	27.7%
	Female	DE0208	3.5%	14.1%	18.0%	21.3%	52.7%
		Model-based	9.5%	14.8%	17.0%	19.5%	29.2%

As mentioned in section 3.1., even when survey data are aggregated over seven years, many LGAs still have insufficient sample size to produce reliable direct estimates. These small sample sizes can produce extreme estimates, which is shown in Table 10. On the other hand, there is very good agreement between model-based and the direct estimated aggregates over 2002–2008 (DE0208) for upper and lower quartiles and median. In all cases the range in model-based estimates indicates that there is considerable variability in the estimates, which is why it is important to be able to report estimates at the LGA level.

Ultimately suitability of the model-based estimates requires the input of a subject matter expert and may depend on the purpose to which the estimates are required.

#### 3.3.5. Comparison of RMSE and RRMSEs of model-based estimates with SE and RSE of direct estimates based on seven years

Table 11 summarizes the SEs and RSEs for LGA-level direct estimates based on 2002–2008 data (DE0208) and RMSE and RRMSEs of model based EBP estimates using the final models, as indicated in Table 9. For the model-based estimates the median RMSE ranges between 3.3% and 6.5% and the maximum varies between 5.2% and 11.3%. The median SE of DE0208 estimates are higher than the median RMSEs of the model-based estimates, with the exception of HDIFF (Table 11). In all cases the maximum RMSEs of the model-based estimates are appreciably smaller than the maximum SE for the DE0208. This shows that the model-based approach has greater impact on LGAs with higher SEs.

**Table 11.** Median and maximum SE and RSE of direct estimates based on data aggregated between 2002 and 2008 (DE0208), compared with estimated RRMSE and RMSE of final logistic EBP estimates, by outcome, summarised over 3 years of estimates.

	RMSE				RRMSE			
	Male		Female		Male		Female	
	Med	Max	Med	Max	Med	Max	Med	Max
ALC								
DE0208	5.5	15	4.4	15.9	13.9	44.8	14.9	50
EBP	3.0	5.5	3.6	6.4	7.8	19.8	12.8	25.8
BMI								
DE0208	5.8	15.2	5.1	16.1	9.2	29.0	10.9	35.7
EBP	4.0	6.0	4.0	5.2	6.8	11.7	8.6	12.5
HDIFF								
DE0208	4.2	16.7	4.0	17.7	25.1	102.1	17.9	51.5
EBP	5.4	9.9	6.5	11.3	27.9	40.1	24.5	41.5
SMK								
DE0208	4.4	15.6	3.3	14	23.1	96.4	20.8	86.6
EBP	3.2	9.1	3.7	7.7	17.5	33.1	23.1	40.2

Note: EBP results are for final model, as indicated in Table 9.

The maximum RRMSEs are always considerably lower for the model-based estimates than direct estimates based on 7 years' data. The medians RRMSEs are lower than the RSEs of the aggregated direct estimates, with the exception of HDIFF, and SMK for female only. For the model-based SAEs the median RRMSE is less than 25% for all except HDIFF for males, where it is 27.9%. Maximum RRMSEs exceed 25% slightly for ALC for females and are more than 25% also for SMK and HDIFF for both males and females. None of the RRMSEs exceed 50%, whereas the maximum RSEs are over 50% for SMK and HDIFF. In all cases the maximum RRMSEs are lower than for the RSEs of the aggregated direct estimates.

If the ABS criteria were used [12], some of the model-based estimates would be flagged for caution, however none would be suppressed.

Whereas multiple years of data are required to publish direct estimates for all LGAs, annual model-based estimates could be published for all LGAs, including for out-of-sample areas and areas where direct estimates would not be acceptable due to small sample sizes or a high RSE. Model-based methods were able to create unbiased results compared with the direct estimates at LGA level. These estimates also agreed with direct estimates when aggregated to higher levels of geography.

The most acceptable covariate specification differed between the outcome variables, so it is necessary to undertake model building for each outcome variable separately.

## 4. Conclusions

Small area estimation methods exploit relationships between the outcome variable and covariates [5] to reduce the level of unexplained between area variability. This study demonstrates a practical development and application of small area estimation methods where there are no strongly correlated covariates at the individual level. The Empirical Best predictor, with appropriate covariates, provided usable model-based estimates based on one year of data for the outcome variables tested in this study. The model based LGA estimates based on a single year of data show considerably improved median and maximum RMSEs and RRMSEs compared with direct estimates based on a single year, and appreciable improvements when compared with direct estimates aggregated over 7 years. They are also unbiased.

The main conclusions are as follows:

**It is necessary to go past a simple null or age model:** The null and age-only models were found to be inappropriate in the assessment of bias at the aggregated level, whereas models with more covariates were able to create unbiased results. Other researchers have found that models either require a spatial random area level term [26] or a model with more covariates included [27]. We did not include a spatial random area-level term; Lawson et al. [28] have noted that any random area-level term is a proxy for covariates that are not in the model.

**Model-based estimates are sufficiently reliable:** The final covariate specification chosen for each outcome variables led to model-based estimates with median (max) RMSE for the four variables by gender of between 3.0% (5.2%) and 5.5% (11.3%) and median (max) RRMSE of between 6.8% (11.7%) and 24.5% (41.5%). Three out of the four outcome variables fulfilled an aspirational goal that the maximum RMSE be less than 10%, with the fourth outcome variable (HDFF) having maximum RMSEs of less than 11.3%. These were appreciable improvements on the maximum RMSEs for the direct estimates based on the 7 years of data.

An important step in acceptance of model-based estimates is to assess them against direct estimates using methods of assessment that take into account that there is no gold standard available. The model-based estimates are generally consistent with direct estimates when aggregated to higher geographic levels. The use of methods to test bias by aggregating to levels where direct estimates have reasonable precision was very useful in this situation, where the parameters are unknown and direct estimates too inaccurate to use methods such as the average empirical bias. The bias test at the LGA level used the synthetic estimator as suggested by Brown et al. [23] and the comparison at aggregated levels compared aggregated EBP.

At the AHS level at least 94% of estimates lie within the 95% confidence interval around the direct estimates for all the outcome variables when the more complex model specifications are used. Whilst at least 90% of aggregated estimates for ALC and BMI lie within the 95% confidence interval around the direct estimates at the quintile of IRSD level, only between 70% and 80% of aggregated model-based estimates for SMK and HDIFF are located within these 95% confidence limits. These two outcome variables have the lower prevalence rates. Creating modelled estimates for outcomes with low prevalence is known to be more difficult than when the prevalence is higher [29]. Even though the aggregated model-based estimates have lower proportions within the 95% confidence intervals than desired for SMK, Hindmarsh [15] shows the trend associated with socioeconomic status is as expected, with smoking rates increasing with increasing disadvantage, based on quintile of IRSD. The null and age models did not have as great a socioeconomic gradient as the more complex models. This approach to the assessment of bias is conservative since it did not take into account the RMSE of the model-based estimate.

For all but two occasions the suggested model created unbiased estimates at the LGA level when compared with the direct estimates. The ONS specification in 2008 for BMI estimates in males is biased, however when the bias test is applied to weighted results it was unbiased; the Global model for SMK in females was biased in 2006. Even if estimates are slightly biased, the consistency at the aggregated level with direct estimates means that, given the lack of other information available about these outcome variables at the small area level, their publication and use will be of great benefit.

**Model-based SAEs can be implemented easily:** The model-based estimates and the associated RMSEs were created using standard procedures in SAS, which allows them to be easily added to the current analytical processes developed for routine reporting.

**RMSEs can be estimated:** The estimated RMSE produced by the SAS GLIMMIX procedure includes the major error components for the EBP-based estimator. Although there are algorithms that include additional terms, those methods add a level of complexity and prevent the methods from being easily implemented. Using RMSEs created by the GLIMMIX procedure opens up small area estimation to more practical application. In the case of the NSW PHS, the routine publication of model-based estimates at the LGA level would greatly enhance the level of information available about health risk factors at the local area for policy development and evaluation.

**Implementation in practice:** This study provides a practical example of the use of small area estimation methods in the creation of annual estimates at the LGA level from a survey that is not designed to provide results at that level. The estimates and associated estimates of RMSEs were easily produced using SAS, making the process easily implemented within the current analysis process for the NSW health survey. Estimates of the rates of health risk factors at the LGA level would provide policy makers with greater confidence in their decisions, compared with using estimates over a longer period of time or much larger geographical areas.

Real data do not respond as perfectly as simulated data. We suggest that the assessment of bias in the absence of a gold standard, as presented in this paper, provides a practical alternative to the use of simulated data. It would strengthen the use of the model-based estimates if any report in which they are presented is accompanied by metadata summarising the bias assessment.

The advantage of the empirical best estimates is that the estimates provide unbiased estimates in LGAs that are not sampled, or where sample size is insufficient to provide reasonable direct survey estimates, and still the estimates approach the direct survey estimates as sample size increases.

The model-based small area estimation method worked best in estimating prevalence of risk alcohol drinking, regular smoking and overweight or obese the three outcomes measuring health risk factors. It also created plausible estimates for the proportion having difficulty getting health care when needed (HDIFF). HDIFF had the largest intraclass correlation of the four outcomes assessed and was the only one with a statistically significant effect of remoteness. This is not surprising, as this outcome variable is likely to depend on the local characteristics of the health system. Inclusion of measures of the availability of health services at the LGA level could improve estimates.

An obvious extension of this work would be to model the rates at the small area level over time. This was considered beyond the scope of the current work.

This paper deliberately used a frequentist approach in order to show that SAE methods can provide reasonable estimates using similar methods to those used for direct survey methodology. Zhang et al. use a similar process [30]. Loux presented a similar concept under the title of Multilevel regressions with poststratification for local estimation at a recent statistical meeting [31].

Bayesian SAE methods [32],[33] are available and can have some advantages over frequentist methods in some situation. For instance, it is possible to include a spatial random effect as well as the area random effect [28]. In addition, it is easy to obtain proportions above a particular value using information from the posterior distribution. However, a higher level of statistical sophistication is required to implement Bayesian methods, particularly the specification of Bayesian priors when sample sizes are small [34]. We would encourage considering fully Hierarchical Bayes methods in environments where Bayesian methods are well understood, and considered robust and easy to implement. This paper shows that frequentist methods produce reasonable small area estimates.
