# Supplementary material for: Creating local estimates from a population health survey: practical application of small area estimation methods
Source: AIMS Public Health. 2020 Jun 22;7(2):403–24. doi: 10.3934/publichealth.2020034 (PMC7327397; doi:10.3934/publichealth.2020034)
Supplement: Supplementary file 1 [file publichealth-07-02-034-s001.pdf]

AIMS Public Health

http://www.aimspress.com/journal/aimsph

AIMS Public Health, 7(2): 403–424.
DOI: 10.3934/publichealth.2020034
Received: 08 June 2020
Accepted: 16 June 2020
Published: 22 June 2020

Research article

Creating local estimates from a population health survey: practical application of small area estimation methods

Diane Hindmarsh[1,2,]* and David Steel[2]

[1] Bureau of Health Information, Level 2, 1 Reserve Road St Leonards, NSW, Australia
[2] National Institute for Applied Statistics Research Australia, University of Wollongong, Wollongong, NSW, Australia

* **Correspondence:** Email: diane.hindmarsh@uow.edu.au; Tel: +61418546442.

Supplementary

1. Calculating the small area estimates using the GLIMMIX procedure in SAS

Table S1. Snippet from dataset showing the way that the survey data and population data are both included in the dataset used for SAE methods in SAS.

obs_no	lga	alcohol1	agecat1 (16–24)	agecat2 (25–34)	agecat3 (35–44)	agecat4 (45–54)	agecat5 (55–64)	agecat6 (65–74)	⋯
1	10050	0	0	0	0	0	0	0	⋯
2	10050	0	0	0	0	0	0	1	⋯
3	10050	1	1	0	0	0	0	0	⋯
4	10050	0	0	0	0	0	0	1	⋯
5	10050	0	0	0	0	1	0	0	⋯
⋮	⋮	⋮	⋮	⋮	⋮	⋮	⋮	⋮	⋯
	10050	.	0.17991	0.15824	0.17021	0.18062	0.13143	0.08597	⋯
⋮	⋮	⋮	⋮	⋮	⋮	⋮	⋮	⋮	⋯

The EBP and synthetic logistic estimates, and associated estimated RMSEs can be obtained from the GLIMMIX procedure using the following method. At the end of the unit record dataset,

append the population data for each LGA as shown in Table S1. Note that the sample part of the dataset uses indicator variables for each component of the covariates for each individual, whilst the proportion within that component of the covariate is included for the population part. As usual, there is no need to include the reference level of the covariate.

The code used in SAS is as follows:

```
proc glimmix  data=&dset
              pconv=1E-5
              maxopt=300;
   nloptions  maxit=200
              maxfunc=350
              gconv=1E-6;
      class &classvars;
         model &depvar = &classvars  / dist = binomial solution;
            random intercept         / subject = LGA solution;
      ods output    covparms = LGT_covparm_RE
                    FitStatistics = LGT_fitstats_RE
                    solutionR=Random_RE;
      output out =  glimmixout pred(blup ilink)     = lgtblup
                             stderr(blup ilink)    = rmse_lgtblup;

run;
```

The output will include estimates and estimated RMSEs for all observations in the input dataset (&dset), including the appended population-based data for each small area. These can be subset by selecting observations that are missing the dependent variable (&depvar).

Supplementary Table A. Covariates included in the Specific (Spec) model, by outcome variable, sex and year.

		Male			Female		
		2006	2007	2008	2006	2007	2008
ALC	Age group	1	1	1	1	1	1
	Health Area	1	1	1	1	1	1
	Birthplace	1	1	1	1	1	1
	Quintile of socio-economic disadvantage				1		
	English spoken at home	1	1	1	1	1	1
	Household size					1	1
	Job	1	1	1	1	1	1
	Marital status				1		1
	Pension						1
	Qualifications	1	1		1	1	1

Continued on next page.

		Male			Female		
		2006	2007	2008	2006	2007	2008
BMI	Age group	1	1	1	1	1	1
	Health Area		1		1	1	1
	Birthplace	1	1		1		1
	Quintile of socio-economic disadvantage	1		1	1	1	1
	English spoken at home			1		1	
	Household size		1				
	Job				1	1	1
	Marital status	1		1		1	1
	Pension					1	
	Private Health cover				1		1
	Qualifications		1			1	
HDIFF	Age group	1	1	1	1	1	1
	Health Area	1	1	1	1	1	1
	ARIA (remoteness)	1	1	1	1	1	1
	Quintile of socio-economic disadvantage	1	1		1	1	1
	Job	1					
	Marital status		1			1	
	Private Health cover		1				1
	Qualifications	1	1		1	1	1
SMK	Age group	1	1	1	1	1	1
	Health Area	1	1				1
	ARIA (remoteness)					1	
	Birthplace					1	
	Quintile of socio-economic disadvantage				1	1	1
	English spoken at home				1		
	Marital status	1	1	1	1	1	1
	Pension	1					
	Private Health cover	1	1	1	1	1	1
	Qualifications	1		1	1	1	1

Note: Covariates that are not statistically significant at all for the outcome of interest are omitted from the list.

Supplementary Table B. Intraclass Correlation Coefficient (ICC) for each covariate specification, by outcome variable, sex and year.

		Male			Female		
		2006	2007	2008	2006	2007	2008
BMI	null	0.3%	1.1%	1.3%	1.8%	1.5%	2.1%
	age	0.4%	1.1%	1.2%	1.7%	1.4%	2.5%
	glob	0	0.2%	0.6%	0.3%	0	0.6%
	ONS	0.4%	dnc	1.2%	0.6%	0.1%	0.9%
	comm	0.1%	0.3%	0.4%	0.3%	0	0.6%
	spec	0.3%	0.7%	0.1%	0.4%	0	0.8%
ALC	null	2.5%	1.2%	1.6%	2.5%	2.3%	2.0%
	age	2.9%	1.8%	1.9%	3.4%	3.0%	2.5%
	glob	1.1%	0	0	0.7%	dnc	0.2%
	ONS	1.7%	0	0.8%	1.2%	1.2%	1.0%
	comm	1.0%	0	0.1%	1.5%	0.6%	0.3%
	spec	1.0%	0	0.1%	0.9%	0.6%	0.3%
HDIFF	null	9.7%	10.6%	10.9%	14.3%	12.6%	10.9%
	age	10.0%	11.0%	11.1%	15.0%	13.9%	12.3%
	glob	2.1%	4.5%	2.2%	3.4%	3.1%	4.5%
	ONS	3.3%	5.2%	3.3%	5.4%	4.2%	5.3%
	comm	2.1%	4.0%	1.9%	3.3%	dnc	4.5%
	spec	1.9%	4.1%	1.9%	3.3%	2.9%	4.5%
SMK	null	2.7%	3.8%	1.5%	1.2%	5.7%	3.9%
	age	2.9%	3.6%	1.2%	1.2%	4.9%	4.0%
	glob	2.4%	0	0.4%	0.2%	5.0%	2.1%
	ONS	1.4%	0.8%	0.4%	0	4.4%	2.7%
	comm	1.6%	1.6%	0	0	4.3%	2.3%
	spec	1.4%	0.9%	0	0	4.1%	2.3%

Note: dnc: did not converge; 0 indicates that the random error term was zero for these models.

Supplementary Table C. Maximum and Median estimated RMSE of EBP and logistic synthetic estimates, by sex, covariate specification group and outcome variable.

Sex	Outcome variable	Covariate specification group*	Median		Maximum	
			EBP	Synth	EBP	Synth
Male	BMI	null, age	3.8%	1.0%	4.1%	1.2%
		Other	3.7%	2.9%	4.5%	3.8%
	ALC	null, age	5.5%	1.1%	6.2%	1.3%
		Other	4.0%	3.1%	4.6%	3.7%
	HDIFF	null, age	6.1%	0.9%	11.5%	1.0%
		Other	5.5%	3.1%	9.1%	4.5%
	SMK	null, age	4.4%	1.0%	5.4%	1.1%
		Other	3.2%	2.2%	4.7%	3.2%
Female	BMI	null, age	5.2%	1.0%	5.9%	1.1%
		Other	3.6%	2.7%	4.2%	3.3%
	ALC	null, age	5.2%	0.9%	6.4%	1.1%
		Other	3.9%	2.6%	5.0%	3.5%
	HDIFF	null, age	6.8%	1.1%	14.6%	1.1%
		Other	6.7%	3.4%	10.9%	4.4%
	SMK	null, age	3.9%	0.7%	5.3%	0.9%
		Other	3.5%	1.9%	5.4%	2.8%

Note: * Null, age—averaged over the null and age-only models; Other—averaged over ONC, Specific, Global and Common models.

AIMS Press

© 2020 the Author(s), licensee AIMS Press. This is an open access article distributed under the terms of the Creative Commons Attribution License (http://creativecommons.org/licenses/by/4.0)
